# Plant Proteomic Research 5.0: From Data to Insights

**DOI:** 10.3390/ijms24010258

**Published:** 2022-12-23

**Authors:** Setsuko Komatsu, Michelle L. Colgrave

**Affiliations:** 1 Faculty of Environmental and Information Sciences, Fukui University of Technology, Fukui 910-0028, Japan; 2 CSIRO Agriculture & Food, 306 Carmody Road, Brisbane, QLD 4053, Australia; 3 School of Science, Edith Cowan University, 270 Joondalup Rd., Joondalup, WA 6027, Australia

Proteomics offers one of the best approaches for the functional analysis of the genome, generating detailed information that can be integrated with that obtained by other classic and omics approaches. It, thus, provides deep knowledge and an understanding of diverse proteoforms and different plant processes. Several different generations of proteomic platforms have appeared in the past 25 years. They have been exploited for describing protein profiles, post-translational modifications, and subcellular localization. Despite recent advancements, more emphasis needs to be given to the protein sample preparation protocols, especially for cases with very low abundance, hydrophobicity, and a large molecular weight. The amalgamation of diverse mass-spectrometry techniques complemented with genome-sequence data and modern bioinformatic analysis offers a powerful tool to identify and characterize novel proteins/proteoforms in spatial and temporal resolution and under different environmental conditions. Furthermore, post-translational modifications, subcellular localization, and protein–protein interactions provide deep insight into protein molecular functions. Current proteomic techniques have gained new insights into plant molecular responses to various stresses.

In this light, the present “Plant Proteomic Research 5.0” Special Issue was conceived to address recent advancements, as well as the limitations of current proteomic techniques, to attain new insights into plant-molecular responses to various environmental stresses. In addition, bioinformatic techniques are needed for more confident identification, quantitation, data analysis, and networking, especially with non-model plants. This Issue builds on the previous Issues, “Plant Proteomic Research” [1], “Plant Proteomic Research 2.0” [2], “Plant Proteomic Research 3.0” [3], and “Plant Proteomic Research 4.0” [4]. “Plant Proteomic Research 5.0” contains eight original articles, which use nano-liquid chromatography combined with mass spectrometry to examine a range of plant materials: paper mulberry [5], *Arabidopsis* [6,7], maize [8], ice plant [9], wheat [10], tomato [11], and cottonseed meal [12]. Proteomic techniques are also used in the identification of stress-responsive mechanisms in plants under conditions, such as cold [7,8], salt [9], flood [10], and metal deficiency [11].

Plant growth and development rely on the conversion of light energy into chemical energy in leaves. Wang et al. [5] uncovered the mechanisms of the golden-yellow phenotype of the hybrid paper mulberry plant using iTRAQ-based proteomic analysis. The mutants of hybrid paper mulberry with golden-yellow leaves showed the reduced chlorophyll/carotenoid content and the increased flavonoid content compared with wild-type plants. The differentially accumulated proteins were primarily involved in chlorophyll synthesis, carotenoid metabolism, and photosynthesis, as well as associated with ribosome pathways. These results suggest that plants adapt to environmental conditions by regulating the proteins to minimize the impact of chlorophyll reduction on growth and survival. This study provides a better understanding of the formation mechanism of the golden-yellow leaf phenotype by combining proteomic approaches. On the other hand, plants are sessile organisms forced to adapt to environmental variations recurring in a day–night cycle. Luklová et al. [6] analyzed the proteins of the Arabidopsis mutant genotypes collected in the middle of the day and the middle of the night, including four mutants in the phytochrome and the circadian clock protein. They indicated a prominent role for reacting-oxygen species metabolism and phytohormone cytokinin in the observed regulations. Additionally, they also shed light on the role of the relatively poorly characterized Phytochrome D, pointing to its connection to glutathione metabolism and the regulation of glutathione S-transferases. This research provided a novel insight into the diel regulations with identifying significantly changed proteins in the night–day protein abundance.

To better understand how plants sense and respond to the early temperature drop, Tan et al. [7] performed data-independent acquisition method-based mass spectrometry analysis to profile the proteins and phosphoproteins of Arabidopsis seedlings upon cold stress in a time-course manner. These results summarized cold-responsive phosphoproteins involved in phospholipid signaling, cytoskeleton reorganization, calcium signaling, and mitogen-activated protein kinase cascades. Additionally, cold also limits the growth and yield of maize in temperate regions. To identify early molecular events during cold shock, maize seedlings were treated under cold stress and analyzed using phosphoproteomic technique [8]. Functional enrichment analysis of cold-responsive proteins and phosphoproteins revealed that early cold response in maize is associated with photosynthesis light reaction, spliceosome, endocytosis, and defense response, consistent with similar studies in Arabidopsis. These results showed that maize seedlings rapidly respond to cold shock, providing a comprehensive landscape for the cold-responsive proteins and phosphoproteins in maize seedlings that can be a significant resource to understand how C_4_ plants respond to a sudden temperature drop.

Ice plant, Mesembryanthemum crystallinum, serves as a model for investigating the molecular mechanisms underlying its salt stress response and tolerance. Zhang et al. [9] cloned one of the homeobox transcription factor genes, McHB7, from the ice plant, and overexpressed it in *Arabidopsis*. The results demonstrate that McHB7 can improve photosynthesis, increase leaf chlorophyll content, and affect the tricarboxylic-acid cycle by regulating metabolites and proteins. Moreover, Mc*HB7* modulates the expression of stress-related proteins to scavenge reactive-oxygen species and enhance plant salt tolerance. As another abiotic stress, flooding impairs wheat growth and considerably affects yield productivity worldwide. In the current work, millimeter-wave irradiation notably enhanced wheat growth, even under flooding stress. To explore the protective mechanisms of millimeter-wave irradiation on wheat under flooding, Komatsu et al. [10] performed quantitative proteomics. These results suggest that millimeter-wave irradiation on wheat seeds improves the recovery of plant growth from flooding via the regulation of glycolysis, reactive-oxygen species scavenging, and cell organization. Additionally, millimeter-wave irradiation could promote tolerance against flooding through the regulation of auxin content in wheat.

Iron (Fe) and manganese (Mn) are two essential elements for plants that compete for the same uptake transporters and show conflicting interactions at the regulatory level. To understand the differential response to both metal deficiencies in plants, two proteomic techniques (two-dimensional gel electrophoresis and label-free shotgun) were used for tomato roots grown under Fe or Mn deficiency [11]. The identified proteins showed that both deficiencies provoked a common and intense cell-wall remodeling. However, the response observed for Fe and Mn deficiencies greatly differed in relation to oxidative stress, coumarin production, and the metabolism of protein, nitrogen, and energy. On the other hand, the proteomic technique was used to analyze cottonseed meal (CSM), which is a good source of dietary proteins but is unsuitable for human consumption due to its gossypol content. To unlock its potential, Tan et al. [12] developed a protein extraction process with a gossypol removal treatment to generate CSM protein isolate (CSMPI) with ultra-low gossypol content. Mass spectrometry analysis of various protein fractions obtained from an in vitro digestibility assay helped to establish the digestibility profile of CSM proteins. Several potential allergens in CSMPI were identified using allergenic prediction software. Overall, these results help to navigate and direct the application of CSMPIs as alternative proteins toward nutritive human food application.

Proteomics is a live and very active discipline and it is an important stepping stone on the way to decipher the intricate secrets and complexities of plant systems. New methodologies, approaches, equipment, and applications will continue to evolve. The Guest Editors hope that this Special Issue will provide readers with a framework for understanding plant proteomics and insights into new research directions within this field. Moreover, the guest editors thank all the authors for their contributions and the reviewers for their critical assessments of these articles.
